# Temporal trends in hospitalisation for stroke recurrence following incident hospitalisation for stroke in Scotland

**DOI:** 10.1186/1741-7015-8-23

**Published:** 2010-04-09

**Authors:** James Lewsey, Pardeep S Jhund, Michelle Gillies, Jim WT Chalmers, Adam Redpath, Andrew Briggs, Matthew Walters, Peter Langhorne, Simon Capewell, John JV McMurray, Kate MacIntyre

**Affiliations:** 1 Public Health and Health Policy, University of Glasgow, 1 Lilybank Gardens, Glasgow G12 8RZ, UK; 2 BHF Glasgow Cardiovascular Research Centre, University of Glasgow, 126 University Place, Glasgow G12 8TA, UK; 3 Information Services Division, NHS National Services Scotland, Gyle Square, 1 South Gyle Crescent, Edinburgh EH12 9EB, UK; 4 Cardiovascular and Medical Sciences, University of Glasgow, Gardiner Institute, Western Infirmary, Glasgow G11 6NT, UK; 5 Public Health, University of Liverpool, Whelan Building, Quadrangle, Liverpool L69 3GB, UK

## Abstract

**Background:**

There are few studies that have investigated temporal trends in risk of recurrent stroke. The aim of this study was to examine temporal trends in hospitalisation for stroke recurrence following incident hospitalisation for stroke in Scotland during 1986 to 2001.

**Methods:**

Unadjusted survival analysis of time to first event, hospitalisation for recurrent stroke or death, was undertaken using the cumulative incidence method which takes into account competing risks. Regression on cumulative incidence functions was used to model the temporal trends of first recurrent stroke with adjustment for age, sex, socioeconomic status and comorbidity. Complete five year follow-up was obtained for all patients. Restricted cubic splines were used to determine the best fitting relationship between the survival events and study year.

**Results:**

There were 128,511 incident hospitalisations for stroke in Scotland between 1986 and 2001, 57,351 (45%) in men. A total of 13,835 (10.8%) patients had a recurrent hospitalisation for stroke within five years of their incident hospitalisation. Another 74,220 (57.8%) patients died within five years of their incident hospitalisation without first having a recurrent hospitalisation for stroke. Comparing incident stroke hospitalisations in 2001 with 1986, the adjusted risk of recurrent stroke hospitalisation decreased by 27%, HR = 0.73 95% CI (0.67 to 0.78), and the adjusted risk of death being the first event decreased by 28%, HR = 0.72 (0.70 to 0.75).

**Conclusions:**

Over the 15-year period approximately 1 in 10 patients with an incident hospitalisation for stroke in Scotland went on to have a hospitalisation for recurrent stroke within five years. Approximately 6 in 10 patients died within five years without first having a recurrent stroke hospitalisation. Using hospitalisation and death data from an entire country over a 20-year period we have been able to demonstrate not only an improvement in survival following an incident stroke, but also a reduction in the risk of a recurrent event.

## Background

Stroke is a major public health problem. It remains a leading cause of death worldwide [1], although mortality rates have declined in most Western European countries and the US [2,3]. Improvements in case-fatality have contributed to these decreases in mortality rates [4,5]. As more people survive their first stroke, the study of recurrent events has become increasingly important, particularly as recurrent stroke events are more likely to be disabling or fatal than first strokes [6].

Recurrent stroke has not been investigated as extensively as incident stroke [7]. Moreover, despite improved management, including secondary prevention, no large-scale population studies have looked at temporal trends in risk of recurrent stroke. A methodological challenge of such research is to account for the improvements in case-fatality, with an increasing proportion of people *at risk *of recurrent stroke over calendar time. The aim of this study was therefore to examine the temporal trends in hospitalisation for recurrent stroke following first hospitalization for stroke in Scotland between 1986 and 2001, after taking into account the competing risk of death.

## Methods

The Information Services Division (ISD) of the National Health Service (NHS) in Scotland collects data on all discharges from NHS hospitals using the Scottish Morbidity Record Scheme (SMR). Data from patient case records are used to code up to six diagnoses at the time of discharge according to the World Health Organisation Classification of Diseases (ICD9 prior to 1996, ICD10 post 1996). The term *discharge *includes both live discharges and deaths. These data are routinely linked to information held by the General Register Office for Scotland relating to all deaths.

The following ICD9 and ICD10 codes were used to identify stroke (ICD10 codes are italicised): 430 (Subarachnoid Haemorrhage), 431 (Intracerebral Haemorrhage), 433 (Occlusion and stenosis of precerebral arteries), 434 (Occlusion of cerebral arteries), 436 (Acute, but ill-defined, cerebrovascular disease), *I60 *(Subarachnoid Haemorrhage), *I61 *(Intracerebral Haemorrhage), *I63 *(Cerebral infarction), *I64 *(Stroke, not specified as haemorrhage or infarction). Incident hospitalisation for stroke was defined as a hospitalisation with a principal diagnosis of stroke with no previous hospitalisation (principal or secondary diagnosis) for cerebrovascular disease (ICD9 430 to 434, 436 to 438 and ICD10 I60 to I69) within five years. SMR identifies stroke with an accuracy of 95% when a stroke code is recorded in the principal diagnostic position [8]. Recurrent hospitalisation for stroke was defined as a hospitalisation with a principal diagnosis of stroke subsequent to the incident event and in a new hospital admission not part of the index stay. We identified the following comorbidities from the data: diabetes, cancer, respiratory disease, heart failure, peripheral arterial disease, atrial fibrillation, hypertension, renal failure, coronary heart disease, rheumatic/valvular heart disease, venous thromboembolism, depression, Parkinsonism, dementia, falls and fractures and alcohol misuse. These comorbidities were identified using principal and secondary diagnoses for any previous hospitalisations in the past five years and secondary diagnoses recorded in the incident stroke hospitalisation. Extra detail on how the SMR is used in identifying stroke and comorbidities can be found elsewhere [9]. Socioeconomic status was defined using the Carstairs-Morris index of deprivation [10], an area based measure based on postcode sector of residence. It is based on the variables of overcrowding, unemployment among men, low social class and not having a car from the 1991 census.

Survival analysis of time to first event, hospitalisation for recurrent stroke or death, was undertaken using both the Kaplan-Meier and cumulative incidence methods [11]. In both methods survival time is censored at death after stroke (a competing risk event) but the Kaplan-Meier method ignores the competing event in calculation of survival probabilities whereas the cumulative incidence method takes the competing event into account. When there are competing events it aids interpretation if results are shown for both the event of interest and the competing event(s) [12]. For this reason, we show results for both hospitalisation for recurrent stroke and death. Complete five-year follow-up was obtained for all patients. Descriptive statistics of the demographics of the group of individuals hospitalised with incident stroke and unadjusted risk of first events are presented for all years as well as for periods at the start and end of the study period (1986 to 1989 and 1998 to 2001). This allows assessment of how demographics and risks have changed over time. We used regression on the cumulative incidence functions [13] to model the temporal trends of first event with adjustment for age, sex, socioeconomic status and comorbidity. We used restricted cubic splines [14] to determine the best fitting relationship between the survival events and study year. A significance level of 0.05 was used throughout. All analyses were carried out using the statistical package R.

## Results

### Demographics of individuals hospitalised with an incident stroke

There were 128,511 incident hospitalisations for stroke in Scotland between 1986 and 2001, 57,351 (45%) in men. The median age at admission was 74 years (IQR 65 to 81). There was a slight socioeconomic deprivation gradient with 17% of incident hospitalisations occurring in individuals living in areas with the least deprived fifth of deprivation scores and 23% occurring in individuals living in areas with the most deprived fifth of deprivation scores (Table 1). The most prevalent comorbidities were coronary heart disease (17%), essential hypertension (15%), atrial fibrillation (10%) and falls and fracture (9%). The distribution of age remained fairly constant across the 15 years with a slight increase in those aged less than 65 years (23% in 1986 to 1989 compared to 25.5% in 1998 to 2001). The socio-economic deprivation gradient narrowed over time. There was an increase in the prevalence of all comorbidities over time apart from Parkinsonism. In absolute and relative terms the largest increases were observed for essential hypertension (8.9% in 1986 to 1989 compared to 24.5% in 1998 to 2001) and atrial fibrillation (5.9% compared to 14.7%). A large relative increase was observed for alcohol misuse (1.7% compared to 4.4%).

**Table 1:** Demographics of individuals hospitalised with an incident stroke

	All years (1986 to 2001)	1986 to 1989	1998 to 2001
	n = 128,511	n = 30,836	n = 32,277
Age group:			
<65 years	23.9	23.0	25.5
65 to 74 years	26.9	27.6	25.8
75+ years	49.2	49.4	48.7
Women	55.4	56.6	54.0
Socioeconomic status:			
1 (least dep.)	17.0	16.7	17.7
2	19.9	19.9	20.3
3	18.9	18.0	19.6
4	21.1	20.9	20.8
5 (most dep.)	23.1	24.5	21.6
Comorbidities:			
Diabetes	8.3	6.4	10.8
Cancer	6.2	4.5	7.9
Respiratory disease	6.3	4.5	8.1
Heart failure	7.7	6.6	8.2
Peripheral arterial disease	6.2	5.6	6.6
Atrial fibrillation	9.9	5.9	14.7
Essential hypertension	15.2	8.9	24.5
Renal failure	2.2	1.3	3.4
Coronary heart disease	16.6	13.0	19.9
Rheum/valv heart disease	2.2	1.4	3.0
Pulm embolism and DVT	2.1	1.7	2.5
Depression	1.6	1.0	2.1
Parkinsonism	1.3	1.4	1.1
Dementia	3.7	3.0	4.5
Falls and fracture	8.6	7.5	9.4
Alcohol misuse	3.0	1.7	4.4

Note: all values in table are %s

### Unadjusted risk of hospitalisation for recurrent stroke and death

**Figure 1:**
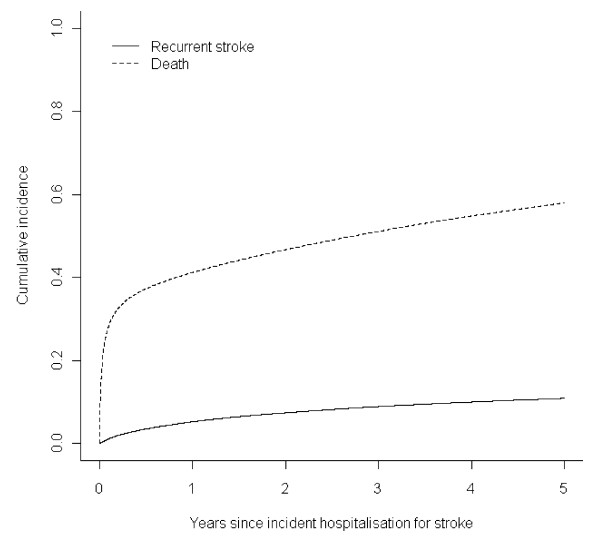
**Cumulative incidence of first events (hospitalisation for recurrent stroke, death) after incident hospitalisation for stroke**.

A comparison of cumulative incidence and Kaplan-Meier estimates for all years can be found in the Appendix (Additional file 1). In the main paper we present cumulative incidence estimates only. Overall 13,835 (10.8%) patients had a recurrent hospitalisation for stroke within five years of their incident hospitalisation. In 1986 to 1989 and 1998 to 2001 the corresponding percentages were 10.8% and 10.6%, respectively. Overall 74,220 (57.8%) patients died within five years of their incident hospitalisation without first having a recurrent hospitalisation for stroke. In 1986 to 1989 and 1998 to 2001 the corresponding percentages were 62.9% and 51.8%, respectively. The cumulative incidence functions of hospitalisation for recurrent stroke and death are plotted in Figure 1. The risk of death was greatest in the first few months following the incident hospitalisation after which followed a more gradual increase out to five years. In comparison, the risk of hospitalisation for recurrent stroke was more constant over the follow-up period.

Table S2 (Additional file 2) shows risks of first events, hospitalisations for recurrent stroke or death, by study year groups and demographics of the incident hospitalisations for stroke. Overall, patients aged 65 to 74 years were at higher risk of recurrent hospitalisation for stroke than those aged <65 years (10.8% vs. 13.1%) but those aged 75 years and above had the lowest risk of recurrent stroke hospitalisation (9.8%). Men were more likely to be hospitalised for recurrent stroke than women (11.6% vs. 10.3%) and those living in areas with the most deprived fifth of deprivation scores were at increased risk compared to those living in areas with the least deprived fifth of deprivation scores (11.4% vs. 10.2%). The comorbidities which had the largest association with risk of recurrent stroke hospitalisation were depression (depression, 18.8%, no depression, 10.8%), essential hypertension (17.0% vs. 9.8%), diabetes (16.6% vs. 10.4%) and atrial fibrillation (15.8% vs. 10.4%).

Overall, increasing age was a strong risk factor for death being the first event after incident hospitalisation for stroke and women were at greater risk than men (60.8% vs. 54.5%). Patients with a comorbidity of heart failure were at greater risk of death than those patients without that comorbidity (76.0% vs. 56.5%). Other comorbidities that were associated with death are cancer, renal failure, essential hypertension, Parkinsonism, dementia and falls and fracture. Age, of course, would influence these associations especially for Parkinsonism, dementia and falls and fracture. Essential hypertension appeared to be protective of death (essential hypertension, 45.2%, no essential hypertension, 60.3%) and the distribution of age was similar in these two groups with those with essential hypertension just a year younger on average.

### Modelling of risk of hospitalisation for recurrent stroke and death

Table 2 shows the unadjusted and adjusted hazard ratios (HRs) from the regressions modelling the cumulative incidence functions of recurrent stroke hospitalisation and death. The unadjusted results show that the risk of both events decreased significantly over time. Comparing 2001 with 1986, the risk of recurrent stroke hospitalisation decreased by 10%, HR = 0.90 95% CI (0.84 to 0.98), and the risk of death being the first event decreased by 30%, HR = 0.70 (0.68 to 0.72). After adjustment, the magnitude of the risk reduction over time for recurrent stroke hospitalisation was increased to over 25%, HR = 0.73 (0.67 to 0.78), whereas it remained approximately the same for risk of death.

**Table 2:** Hazard ratios for hospitalisation for recurrent stroke and for death

	HRs for hospitalisation for recurrent stroke		HRs for death	
	unadjusted	adjusted	unadjusted	adjusted
Year of study:				
1991	0.95 (0.89 to 1.02)	0.93 (0.86 to 0.99)	0.94 (0.92 to 0.97)	0.92 (0.90 to 0.95)
1996	0.99 (0.94 to 1.05)	0.89 (0.84 to 0.94)	0.80 (0.79 to 0.82)	0.81 (0.79 to 0.83)
2001	0.90 (0.84 to 0.98)	0.73 (0.67 to 0.78)	0.70 (0.68 to 0.72)	0.72 (0.70 to 0.75)
Age group:				
65 to 74 years		1.17 (1.12 to 1.22)		1.84 (1.79 to 1.89)
75+ years		0.90 (0.86 to 0.95)		2.97 (2.90 to 3.04)
Sex: Women		0.92 (0.89 to 0.95)		1.04 (1.02 to 1.05)
Socioecon. status:				
2		1.09 (1.03 to 1.16)		1.03 (1.01 to 1.06)
3		1.04 (0.98 to 1.10)		1.06 (1.04 to 1.09)
4		1.03 (0.97 to 1.09)		1.07 (1.05 to 1.10)
5 (most dep.)		1.09 (1.03 to 1.15)		1.07 (1.05 to 1.10)
Comorbidities:				
Diabetes		1.42 (1.35 to 1.50)		0.99 (0.97 to 1.02)
Cancer		0.95 (0.89 to 1.02)		1.37 (1.34 to 1.41)
Respiratory disease		1.07 (1.00 to 1.14)		1.14 (1.11 to 1.18)
Heart failure		0.84 (0.79 to 0.90)		1.42 (1.38 to 1.45)
Peripheral arterial disease		1.16 (1.09 to 1.23)		1.17 (1.14 to 1.20)
Atrial fibrillation		1.49 (1.41 to 1.56)		0.91 (0.89 to 0.93)
Essential hypertension		1.65 (1.58 to 1.72)		0.70 (0.69 to 0.72)
Renal failure		0.94 (0.84 to 1.05)		1.45 (1.38 to 1.52)
Coronary heart disease		1.18 (1.13 to 1.24)		1.03 (1.01 to 1.06)
Rheum/valv heart disease		1.13 (1.02 to 1.26)		0.96 (0.92 to 1.01)
Pulm embolism and DVT		1.17 (1.05 to 1.31)		1.05 (1.00 to 1.10)
Depression		1.69 (1.52 to 1.88)		0.84 (0.79 to 0.89)
Parkinsonism		1.13 (0.98 to 1.30)		1.07 (1.02 to 1.13)
Dementia		1.29 (1.19 to 1.40)		1.09 (1.06 to 1.13)
Falls and fracture		1.00 (0.94 to 1.06)		1.12 (1.10 to 1.15)
Alcohol misuse		1.13 (1.03 to 1.24)		1.15 (1.10 to 1.20)

Note: the reference categories for year of study, age group, sex, deprivation and the comorbidities are 1986, <65 years, men, one (least deprived) and comorbidity not present, respectively; 95% CIs in parentheses

## Discussion

Slightly more than 1 in 10 patients with an incident hospitalisation for stroke in Scotland between 1986 and 2001 went on to have a hospitalisation for recurrent stroke within five years. Approximately 6 in 10 patients died within five years of their incident hospitalisation without first having a recurrent hospitalisation for stroke. The risk of hospitalisation for recurrent stroke within five years was 27% lower for patients who had their incident stroke hospitalisation in 2001 compared to the corresponding risk for patients who had their incident stroke hospitalisation in 1986, after adjustment for age, sex, socioeconomic status and comorbidity. The corresponding risk reduction for death was 28%.

In previous epidemiological studies of stroke we have reported that the overall incidence of stroke in Scotland has declined substantially between 1986 and 2005 [9]. Indeed, the relative risk reduction in incidence between 2001 and 1986 was 33%, a similar reduction to those above for recurrent events and death. This would imply that primary and secondary prevention strategies for stroke in Scotland have both been effective. Certainly primary prevention for blood pressure and elevated cholesterol has improved steadily over the study period, as has secondary prevention for coronary heart disease [15,16]. Data focussed specifically on trends in stroke patient therapeutics in Scotland are not so easily identified. However, data from the Scottish Stroke Care Audit (SSCA) are available for the end of our study period. For example, they show the percentage of patients with a definite ischaemic event who received aspirin within two days of hospital admission increasing from 43% in 2003/2004 [17] to 57% in 2005 [18] and to 69% in 2007 [18]. Furthermore, we identified the existence and capacity of stroke units during the entire study period by a variety of methods including the SSCA and personal contact through a national network of stroke clinicians. Applying this to the SMR data we show that the percentage of stroke patients in Scotland treated in a hospital with a stroke unit service able to provide for at least 50% of patients has increased steadily (from zero in 1990, to 12% in 1995, 29% in 2000, reaching approximately 69% in 2005).

Adjustment for comorbid diagnoses increased the strength of association between the risk of hospitalisation for recurrent stroke and year of study. This was mostly driven by atrial fibrillation and essential hypertension because the prevalence of these comorbidities increased markedly across the study years and both were strongly associated with risk of recurrent stroke hospitalisation. To illustrate this, if these comorbidities are not adjusted for in the regression model the HR comparing risk of recurrent stroke hospitalisation in 2001 to 1986 is 0.84 rather than 0.73 as reported in Table 2, much closer to the unadjusted estimate of 0.90. Analysis of audit data from ISD would suggest that there has been a real increase in the prevalence of comorbid diagnoses over time (personal communication with Adam Redpath) and that these findings are likely to reflect both a real increase in comorbid disease as well as more active recognition and diagnosis of these conditions.

There are limitations to our study. First, we have used discharge codes to identify our study cohort. Internal validation studies of the Scottish Morbidity Record scheme have shown the data to identify stroke with an accuracy of 95% when recorded in a principal diagnostic position. However, the accuracy of coding is likely to vary on an institutional basis. Also, we have used three digit ICD codes to identify stroke. However, in ICD9 the fifth digit is used to identify presence or absence of cerebral infarction. The codes that are affected by this (433 and 434) only account for a small percentage of all strokes in our study and so the bias of including patients without cerebral infarction is small. Second, our risk adjustments may be inadequate because of lack of clinical detail, for example regarding the severity of stroke. Furthermore, we could not adjust temporal trends for stroke type because of substantial changes in the relative distribution of subarachnoid haemorrhage, intracerebral haemorrhage, cerebral infarction and 'type not specified' due to improvements in availability and use of imaging.

The main strengths of our study are that we have studied a whole population (5.1 million people) over a 20-year period and have obtained complete five-year follow-up. This is the largest study of recurrent stroke to date. In addition we have used a methodology that has allowed us to examine the two endpoints of recurrent stroke and death simultaneously, which is appropriate for a condition such as stroke which is associated with a high case-fatality.

There are few studies that have investigated temporal trends in risk of recurrent stroke. A community-based study from Australia [19] compared survival and recurrent stroke over five years after first-ever stroke, for patients enrolled in 1989 to 1990 and 1995 to 1996. There was no improvement in five-year survival between the two periods but a considerable reduction in risk of recurrent stroke was observed (32% at five years for 1989 to 1990 cohort, compared to 23% at five years for 1995 to 1996 cohort). Unfortunately, due to a small sample size this result was not found statistically significant. There have been a number of other small community-based studies of recurrent stroke in the past 25 years. They report rates of recurrent stroke of 7% [20] and 9% [21], at one year; 9% [22] and 10.5% [23], at two years; 15% [24], 16% [20] and 20% [25], at five years; 17% [26] and 24.5% [20] at 10 years. There have been fewer studies of recurrent stroke based on hospital discharges. A Canadian study [27] reported a rate of recurrent stroke of 6 to 10% at one year depending on stroke type, and two independent US studies report rates of 12% [28] at two years and 17% [29] at five years. There is a lack of consistency in these findings and this is due at least in part to differences in definition of recurrent stroke (for example, including or excluding recurrent strokes in the first few weeks after the incident stroke, or including only patients discharged alive). Nevertheless, our Kaplan-Meier estimate of recurrent stroke hospitalisations at five years (19.6%, reported separately for men and women in Table S1, Additional file 1) is comparable to the literature.

## Conclusions

Using hospitalisation and death data from an entire country over a 20-year period we were able to demonstrate not only an improvement in survival following an incident stroke, but also a reduction in the risk of a recurrent event. The implications of our study are that improvements in the management of stroke and greater emphasis on secondary prevention have improved outcomes following an incident hospitalisation for stroke.

## Abbreviations

CI: Confidence Interval; HR: Hazard Ratio; ICD: International Classification of Diseases; IQR: Inter-Quartile Range; ISD: Information Services Division; NHS: National Health Service; SMR: Scottish Morbidity Record Scheme; SSCA: Scottish Stroke Care Audit

## Competing interests

The authors declare that they have no competing interests.

## Authors' contributions

JL planned the analyses, conducted the analyses and drafted the manuscript. PJ, JC, AR, MW, SC, JM and KM helped design the study, assisted in the planning of the analyses, and provided critical input during the drafting of the paper. MG, AB and PL assisted in the planning of the analyses and provided critical input during the drafting of the paper. All authors read and approved the final manuscript.

## Pre-publication history

The pre-publication history for this paper can be accessed here:

http://www.biomedcentral.com/1741-7015/8/23/prepub

## Supplementary Material

Additional file 1 **Appendix and Table S1**. A comparison of cumulative incidence and Kaplan-Meier estimates for all years Click here for file

Additional file 2 **Table S2**. Risk of first events (hospitalisation for recurrent stroke at five years, death at five years) after incident hospitalisation for stroke Click here for file
