# Supplementary material for: Temporal trends in hospitalisation for stroke recurrence following incident hospitalisation for stroke in Scotland
Source: BMC Med. 2010 Apr 9;8:23. doi: 10.1186/1741-7015-8-23 (PMC2859404; doi:10.1186/1741-7015-8-23)
Supplement: Additional file 1 — Appendix and Table S1. A comparison of cumulative incidence and Kaplan-Meier estimates for all years [file 1741-7015-8-23-S1.DOC]

**Appendix**

The cumulative incidence estimates reported in this paper can be thought of as the percentage of events that occur taking into account time to event and censoring. In this study there is no censoring and so the percentages of recurrent stroke events are very similar to the estimates in Table S2, Additional file 2 (e.g., across all years for patients aged less than 65 years 3,266 (10.6%) had recurrent stroke hospitalisation within 5 years – very similar to corresponding estimate in Table S2, 10.8%).

Table S1 shows the Kaplan-Meier estimates of hospitalisation for recurrent stroke and death within 5 years. The Kaplan-Meier estimates are higher than the corresponding cumulative incidence estimates in Table S2, especially for hospitalisation for recurrent stroke. The reason for these differences are that the Kaplan-Meier estimator is biased for survival analysis of competing risks and overestimates the probability of failure, and the bias is greater when the risk of the competing events is larger [1]. The bias is due to informative censoring – the Kaplan-Meier estimator assumes that patients with censored survival times due to a competing event occurring have, on average, the same risk of experiencing the event of interest as those patients who are not censored. Clearly this is not the case in this study – a patient who has a censored survival time due to death cannot experience a hospitalisation for recurrent stroke.

1. Putter H, Fiocco M, Geskus RB: **Tutorial in biostatistics: competing risks and multi-state models.** *Statistics in Medicine* 2007, **26**:2389-430.

**Table S1: Risk of hospitalisation for recurrent stroke and risk of death** at 5 years after incident hospitalisation for stroke

|  | **All years (1986-2001)** | |
| --- | --- | --- |
|  | **Recurrent stroke** | **Death** |
| Age group < 65 | 14.3 (13.8-14.7) | 32.1 (31.5-32.6) |
| (years) 65-74 | 21.8 (21.2-22.4) | 52.7 (52.1-53.2) |
| 75+ | 22.9 (22.4-23.5) | 73.4 (73.1-73.8) |
| Men | 19.5 (19.1-20.0) | 54.5 (54.1-55.0) |
| Women | 19.6 (19.1-20.0) | 60.8 (60.4-61.1) |
| Socioecon. 1 (least dep.) | 18.3 (17.6-19.0) | 58.2 (57.6-58.9) |
| status 2 | 20.4 (19.7-21.1) | 58.5 (57.9-59.1) |
| 3 | 19.5 (18.8-20.2) | 58.2 (57.6-58.7) |
| 4 | 19.2 (18.5-19.9) | 58.1 (57.5-58.7) |
| 5 (most dep.) | 20.1 (19.5-20.8) | 57.1 (56.5-57.6) |
| Comorbidities: |  |  |
| No diabetes | 18.6 (18.3-18.9) | 58.1 (57.8-58.4) |
| Diabetes | 29.3 (28.1-30.5) | 56.9 (55.9-57.8) |
| No cancer | 19.3 (19.0-19.6) | 57.0 (56.7-57.2) |
| Cancer | 26.2 (24.5-27.9) | 73.5 (72.6-74.5) |
| No respiratory disease | 19.2 (18.9-19.5) | 57.5 (57.2-57.8) |
| Respiratory disease | 25.9 (24.5-27.4) | 65.6 (64.6-66.7) |
| No heart failure | 18.9 (18.6-19.2) | 56.5 (56.2-56.8) |
| Heart failure | 31.7 (30.0-33.4) | 76.0 (75.1-76.8) |
| No peripheral arterial diseas | 19.0 (18.7-19.3) | 57.6 (57.3-57.9) |
| Peripheral arterial disease | 28.4 (27.0-30.0) | 64.1 (63.0-65.1) |
| No atrial fibrillation | 18.4 (18.1-18.7) | 57.6 (57.3-57.9) |
| Atrial fibrillation | 31.0 (29.8-32.2) | 61.4 (60.6-62.3) |
| No essential hypertension | 18.1 (17.8-18.5) | 60.3 (60.0-60.6) |
| Essential hypertension | 25.5 (24.8-26.3) | 45.2 (44.5-45.9) |
| No renal failure | 19.4 (19.1-19.7) | 57.6 (57.4-57.9) |
| Renal failure | 32.8 (29.8-36.1) | 73.5 (71.8-75.1) |
| No coronary heart disease | 18.1 (17.8-18.5) | 57.3 (57.0-57.6) |
| Coronary heart disease | 27.2 (26.3-28.1) | 61.6 (60.9-62.3) |
| No rheum/valv heart disease | 19.4 (19.1-19.7) | 58.0 (57.7-58.3) |
| Rheum/valv heart disease | 26.2 (23.9-28.6) | 58.6 (56.7-60.4) |
| No pulm embolism and DVT | 19.4 (19.1-19.7) | 57.9 (57.6-58.2) |
| Pulm embolism and DVT | 25.7 (23.4-28.2) | 61.3 (59.4-63.2) |
| No depression | 19.3 (19.0-19.7) | 58.0 (57.8-58.3) |
| Depression | 31.3 (28.6-34.1) | 55.6 (53.4-57.8) |
| No parkinsonism | 19.5 (19.2-19.8) | 57.8 (57.5-58.0) |
| Parkinsonism | 30.3 (26.5-34.5) | 74.9 (72.7-77.0) |
| No dementia | 19.2 (18.9-19.5) | 57.3 (57.0-57.6) |
| Dementia | 33.4 (31.0-36.0) | 75.9 (74.7-77.1) |
| No falls and fracture | 19.3 (19.0-19.6) | 56.8 (56.5-57.1) |
| Falls and fracture | 23.5 (22.3-24.8) | 70.7 (69.8-71.6) |
| No alcohol misuse | 19.5 (19.2-19.8) | 58.2 (57.9-58.4) |
| Alcohol misuse | 21.3 (19.6-23.0) | 52.6 (51.0-54.1) |

Note: 95% CIs in parentheses
