# Supplementary material for: Temporal trends in hospitalisation for stroke recurrence following incident hospitalisation for stroke in Scotland
Source: BMC Med. 2010 Apr 9;8:23. doi: 10.1186/1741-7015-8-23 (PMC2859404; doi:10.1186/1741-7015-8-23)
Supplement: Additional file 2 — Table S2. Risk of first events (hospitalisation for recurrent stroke at five years, death at five years) after incident hospitalisation for stroke [file 1741-7015-8-23-S2.DOC]

**Table S2: Risk of first events (hospitalisation for recurrent stroke at 5 years, death at 5 years) after incident hospitalisation for stroke**

|  | **All years (1986-2001)** | | **1986-1989** | | **1998-2001** | |
| --- | --- | --- | --- | --- | --- | --- |
|  | **Recurrent stroke** | **Death** | **Recurrent stroke** | **Death** | **Recurrent stroke** | **Death** |
| Age group < 65 | 10.8 (10.4-11.1) | 32.1 (31.5-32.6) | 11.1 (10.4-11.9) | 38.6 (37.4-39.7) | 10.4 (9.7-11.0) | 26.5 (25.6-27.5) |
| (years) 65-74 | 13.1 (12.8-13.5) | 52.7 (52.1-53.2) | 13.7 (13.0-14.5) | 59.1 (58.0-60.2) | 12.6 (11.9-13.4) | 45.2 (44.1-46.3) |
| 75+ | 9.8 (9.5-10.0) | 73.4 (73.1-73.8) | 9.5 (9.0-9.9) | 76.5 (75.8-77.2) | 9.9 (9.5-10.4) | 69.2 (68.5-70.0) |
| Men | 11.6 (11.4-11.9) | 54.5 (54.1-55.0) | 11.9 (11.3-12.5) | 59.8 (58.9-60.6) | 11.3 (10.8-11.9) | 48.2 (47.3-49.0) |
| Women | 10.3 (10.1-10.6) | 60.8 (60.4-61.1) | 10.4 (9.9-10.8) | 65.5 (64.7-66.2) | 10.2 (9.8-10.7) | 55.6 (54.9-56.4) |
| Socioecon. 1 (least dep.) | 10.2 (9.8-10.6) | 58.2 (57.6-58.9) | 10.5 (9.6-11.3) | 63.8 (62.5-65.2) | 10.1 (9.3-10.9) | 51.9 (50.5-53.2) |
| status 2 | 11.2 (10.8-11.6) | 58.5 (57.9-59.1) | 11.7 (10.8-12.5) | 63.6 (62.3-64.8) | 10.8 (10.1-11.6) | 52.6 (51.4-53.9) |
| 3 | 10.8 (10.4-11.2) | 58.2 (57.6-58.7) | 10.8 (10.0-11.7) | 62.4 (61.1-63.8) | 11.2 (10.5-12.0) | 53.1 (51.9-54.4) |
| 4 | 10.7 (10.3-11.1) | 58.1 (57.5-58.7) | 10.7 (10.0-11.5) | 63.5 (62.2-64.7) | 10.8 (10.1-11.6) | 52.3 (51.1-53.5) |
| 5 (most dep.) | 11.4 (11.1-11.8) | 57.1 (56.5-57.6) | 11.3 (10.5-12.0) | 62.0 (60.9-63.1) | 10.7 (10.0-11.4) | 51.1 (49.9-52.3) |
| Comorbidities: |  |  |  |  |  |  |
| No diabetes | 10.4 (10.2-10.6) | 58.1 (57.8-58.4) | 10.7 (10.3-11.0) | 63.0 (62.5-63.6) | 10.0 (9.7-10.4) | 52.4 (51.8-53.0) |
| Diabetes | 16.6 (15.8-17.3) | 56.9 (55.9-57.8) | 16.2 (14.6-17.9) | 62.6 (60.4-64.8) | 16.8 (15.5-18.1) | 50.8 (49.1-52.4) |
| No cancer | 10.9 (10.8-11.1) | 57.0 (56.7-57.2) | 11.0 (10.7-11.4) | 62.3 (61.8-62.9) | 10.7 (10.3-11.0) | 50.7 (50.1-51.2) |
| Cancer | 10.4 (9.8-11.1) | 73.5 (72.6-74.5) | 10.7 (9.0-12.4) | 77.1 (74.8-79.4) | 11.7 (10.4-12.9) | 69.8 (68.0-71.6) |
| No respiratory disease | 10.8 (10.6-11.0) | 57.5 (57.2-57.8) | 10.9 (10.5-11.3) | 62.6 (62.1-63.2) | 10.6 (10.2-10.9) | 51.4 (50.8-52.0) |
| Respiratory disease | 12.8 (12.1-13.5) | 65.6 (64.6-66.7) | 13.5 (11.7-15.4) | 70.9 (68.4-73.3) | 12.4 (11.2-13.7) | 61.4 (59.5-63.3) |
| No heart failure | 10.9 (10.7-11.0) | 56.5 (56.2-56.8) | 11.0 (10.7-11.4) | 61.9 (61.3-62.5) | 10.6 (10.3-11.0) | 50.3 (49.7-50.8) |
| Heart failure | 11.6 (10.9-12.2) | 76.0 (75.1-76.8) | 10.8 (9.4-12.2) | 78.4 (76.5-80.2) | 11.9 (10.7-13.2) | 73.6 (71.9-75.3) |
| No peripheral arterial diseas | 10.7 (10.5-10.9) | 57.6 (57.3-57.9) | 10.8 (10.5-11.2) | 62.7 (62.1-63.3) | 10.4 (10.1-10.8) | 51.7 (51.1-52.2) |
| Peripheral arterial disease | 14.2 (13.5-15.0) | 64.1 (63.0-65.1) | 13.9 (12.3-15.6) | 67.8 (65.6-70.1) | 15.1 (13.6-16.6) | 59.8 (57.7-61.9) |
| No atrial fibrillation | 10.4 (10.2-10.5) | 57.6 (57.3-57.9) | 10.8 (10.4-11.1) | 62.9 (62.3-63.4) | 10.1 (9.7-10.4) | 50.7 (50.1-51.3) |
| Atrial fibrillation | 15.8 (15.2-16.4) | 61.4 (60.6-62.3) | 15.3 (13.6-17.0) | 65.2 (63.0-67.5) | 14.7 (13.6-15.7) | 60.9 (59.5-62.3) |
| No essential hypertension | 9.8 (9.6-10.0) | 60.3 (60.0-60.6) | 10.5 (10.1-10.8) | 64.4 (63.8-64.9) | 8.8 (8.4-9.2) | 55.1 (54.5-55.7) |
| Essential hypertension | 17.0 (16.5-17.5) | 45.2 (44.5-45.9) | 16.7 (15.3-18.1) | 49.5 (47.5-51.4) | 16.7 (15.9-17.6) | 43.3 (42.2-44.4) |
| No renal failure | 10.9 (10.7-11.1) | 57.6 (57.4-57.9) | 11.0 (10.7-11.4) | 62.8 (62.3-63.4) | 10.7 (10.4-11.1) | 51.5 (50.9-52.0) |
| Renal failure | 12.2 (10.9-13.4) | 73.5 (71.8-75.1) | 11.5 (8.3-14.8) | 74.8 (70.4-79.2) | 11.6 (9.7-13.5) | 72.7 (70.0-75.3) |
| No coronary heart disease | 10.3 (10.1-10.5) | 57.3 (57.0-57.6) | 10.6 (10.2-11.0) | 62.4 (61.8-63.0) | 9.9 (9.5-10.2) | 50.8 (50.2-51.4) |
| Coronary heart disease | 14.0 (13.5-14.5) | 61.6 (60.9-62.3) | 13.6 (12.6-14.7) | 67.2 (65.7-68.7) | 14.2 (13.4-15.1) | 57.8 (56.6-59.1) |
| No rheum/valv heart disease | 10.8 (10.7-11.0) | 58.0 (57.7-58.3) | 11.0 (10.6-11.3) | 63.1 (62.5-63.6) | 10.7 (10.3-11.0) | 52.0 (51.4-52.5) |
| Rheum/valv heart disease | 14.3 (12.9-15.6) | 58.6 (56.7-60.4) | 16.0 (12.4-19.5) | 59.3 (54.6-64.0) | 13.4 (11.3-15.6) | 60.3 (57.2-63.4) |
| No pulm embolism and DVT | 10.9 (10.7-11.0) | 57.9 (57.6-58.2) | 11.0 (10.6-11.4) | 62.9 (62.4-63.5) | 10.7 (10.3-11.0) | 52.1 (51.5-52.6) |
| Pulm embolism and DVT | 13.4 (12.1-14.7) | 61.3 (59.4-63.2) | 11.6 (8.8-14.4) | 67.1 (63.0-71.3) | 13.4 (11.1-15.8) | 57.4 (54.0-60.9) |
| No depression | 10.8 (10.6-11.0) | 58.0 (57.8-58.3) | 10.9 (10.6-11.3) | 63.1 (62.5-63.6) | 10.6 (10.3-11.0) | 52.2 (51.6-52.7) |
| Depression | 18.8 (17.0-20.5) | 55.6 (53.4-57.8) | 19.6 (15.1-24.1) | 56.8 (51.1-62.4) | 16.5 (13.7-19.3) | 54.1 (50.3-57.8) |
| No parkinsonism | 10.9 (10.7-11.1) | 57.8 (57.5-58.0) | 11.0 (10.6-11.4) | 62.8 (62.3-63.4) | 10.7 (10.4-11.0) | 52.0 (51.4-52.5) |
| Parkinsonism | 12.5 (10.8-14.1) | 74.9 (72.7-77.0) | 12.1 (8.9-15.2) | 74.4 (70.1-78.6) | 14.9 (11.1-18.7) | 74.0 (69.3-78.6) |
| No dementia | 10.8 (10.7-11.0) | 57.3 (57.0-57.6) | 11.0 (10.6-11.3) | 62.6 (62.1-63.2) | 10.7 (10.3-11.0) | 51.0 (50.4-51.6) |
| Dementia | 13.1 (12.1-14.0) | 75.9 (74.7-77.1) | 12.9 (10.6-15.1) | 74.8 (71.9-77.7) | 12.2 (10.5-13.9) | 77.4 (75.2-79.5) |
| No falls and fracture | 10.9 (10.8-11.1) | 56.8 (56.5-57.1) | 11.1 (10.7-11.5) | 62.2 (61.6-62.8) | 10.8 (10.4-11.1) | 50.5 (49.9-51.1) |
| Falls and fracture | 10.6 (10.0-11.2) | 70.7 (69.8-71.6) | 10.4 (9.1-11.7) | 72.4 (70.5-74.3) | 10.7 (9.6-11.8) | 68.3 (66.6-70.0) |
| No alcohol misuse | 10.8 (10.7-11.0) | 58.2 (57.9-58.4) | 11.0 (10.6-11.3) | 63.2 (62.6-63.8) | 10.7 (10.3-11.0) | 52.3 (51.7-52.9) |
| Alcohol misuse | 13.2 (12.1-14.2) | 52.6 (51.0-54.1) | 13.6 (10.6-16.6) | 51.9 (47.6-56.3) | 12.0 (10.3-13.7) | 50.0 (47.4-52.6) |

Note: 95% CIs in parentheses
